# Sol-gel synthesis of α-Al_2_O_3_ with enhanced porosity via dicarboxylic acid templating

**DOI:** 10.1038/s41598-019-56294-1

**Published:** 2019-12-27

**Authors:** Simon Carstens, Christian Splith, Dirk Enke

**Affiliations:** Institute of Chemical Technology, Universität Leipzig, Linnéstraße 3, 04103 Leipzig, Germany

**Keywords:** Ceramics, Materials chemistry

## Abstract

One of the major routes to synthesize macroporous α-Al_2_O_3_ is the sol-gel process in presence of templates. Templates include polymers as well as carboxylic acids, such as citric acid. By careful choice of the template, pore diameters can be adjusted between 110 nm and several µm. We report the successful establishment of plain short-chain dicarboxylic acids (DCA) as porogenes in the sol-gel synthesis of macroporous α-Al_2_O_3_. By this extension of the recently developed synthesis route, a very precise control of pore diameters is achieved, in addition to enhanced macropore volumes in α-Al_2_O_3_. The formation mechanism thereof is closely related to the one postulated for citric acid, as thermal analyses show. However, since branching in the DCA-linked alumina nuclei is not possible, close monomodal pore width distributions are attained, which are accompanied by enhanced pore volumes. This is a significant improvement in terms of controlled enhanced porosity in the synthesis of macroporous α-Al_2_O_3_.

## Introduction

Macroporous α-alumina is a unique material, combining the refractoriness and chemically inert qualities of pure corundum with the advantages of porous materials having an elevated specific surface area. This includes among others adsorptive properties^1–3^, and facilitated mass transfer in combination with thermal stability, which is an essential feature for certain catalyst supports^4,5^.

Macroporous α-Al_2_O_3_ can be obtained via different routes, e.g., transformation of diaspore^6,7^, hydrothermal methods^8,9^, or anodic oxidation of aluminum chips and their successive calcination to porous alumina membranes^10,11^. Yet one of the most versatile methods is found in the sol-gel synthesis in presence of templates, starting from aluminum alkoxides^12^ or aluminum salts^13–15^. We recently reported a variation of the well-established PEO-templated synthesis with epoxide-mediated gelation in which we replaced the costly polymer by citric acid – a green, inexpensive, and readily available template^16^.

This article discloses a further improvement of the citric acid-assisted route, using dicarboxylic acids (DCA) as porogenes. The mechanism is closely related to the one investigated for citric acid, as thermal analyses show. However, due to their simpler chemical structure with only two carboxylic acid functional groups and linear composition, oxalic acid and its homologues enable a much more precise control of the pore diameter in the macropore range, along with enhanced pore volumes.

## Experimental Section

### Reagents

All reagents were used as received, without further purification or treatment. AlCl_3_·6H_2_O (99% purity) was purchased from Alfa Aesar. Propylene oxide was delivered by Acros Organics, citric acid (food quality) by purux, and all employed dicarboxylic acids by Merck. Solvents (ethanol and distilled water) were taken from domestic lines.

### Synthesis of macroporous α-Al_2_O_3_

For a standard procedure, AlCl_3_·6H_2_O and distilled water were placed in the reaction vessel and dissolved in ethanol. The respective dicarboxylic acid was added in a molar ratio *φ*_*Al*_ of Al^3+^/DCA = 10 to the mixture and dissolved immediately before the reaction. Additionally, sample C2#2 was prepared using twice the amount of oxalic acid. All compositions are listed in Table 1. Propylene oxide was added with a syringe under vigorous stirring to the cooled reaction mixture. After gel ageing, solvent exchange, and drying, calcination was carried out in air at 1200 °C for 6 h. A detailed description of the complete procedure can be found elsewhere ^16^.

**Table 1:** Composition of all samples, synthesized according to the standard procedure.

Sample	Additive	AlCl_3_·6H_2_O	H_2_O	Ethanol	Propylene oxide	Additive	*φ*_*Al*_^[a]^
*Ref0*^16^	*None*	*7.80 g*	*6.98 g*	*7.9 g*	*7 mL*	—	*n/a*
*CA68*^16^	citric acid*	*7.80 g*	*6.98 g*	*7.9 g*	*7 mL*	*0.68 g*	10
C2#1	oxalic acid**	7.80 g	6.86 g	7.9 g	7 mL	0.41 g**	10
C2#2	oxalic acid**	7.80 g	6.74 g	7.9 g	7 mL	0.82 g**	5
C3	malonic acid	7.80 g	6.98 g	7.9 g	7 mL	0.34 g	10
C4	succinic acid	7.80 g	6.98 g	7.9 g	7 mL	0.38 g	10
C5	glutaric acid	7.80 g	6.98 g	7.9 g	7 mL	0.43 g	10
C6	adipic acid	7.80 g	6.98 g	7.9 g	7 mL	0.47 g	10

^[a]^Ratio Al^3+^/additive.

*Monohydrate **dihydrate.

### Characterization of macroporous α-Al_2_O_3_

The obtained granular material was characterized by mercury intrusion, SEM, nitrogen sorption, and XRD. Furthermore, thermal analyses (TG and DTA) were conducted on as-synthesized, dried samples.

Mercury intrusion was performed on a Pascal 440 porosimeter by ThermoScientific/Porotec with pressures ranging from 0.2 mbar to 4000 bar. Mercury surface tension was assumed to be 0.484 N/m, its contact angle was set to 141.3°. Samples were outgassed at 0.2 mbar for 10 minutes at ambient temperature prior to filling the dilatometer with mercury.

Scanning electron microscopy (SEM) images were obtained using a Leo Gemini 1530 by Zeiss. Samples were fixated on a carbon foil and vapor coated with a gold film. Accelerating voltage was 10 kV. Secondary electrons were collected by an Everhart-Thornley detector.

Nitrogen sorption was carried out on an ASAP 2000, Micromeritics. Prior to examination, the samples were dried, outgassed, and activated at 300 °C under vacuum. Determination of the specific surface area (*A*_*BET*_) was conducted using the linearized form of the BET equation in the range of 0.05 ≤ p/p_0_ ≤ 0.30.

For thermal analyses, 25 mg of non-calcined sample were mixed with 25 mg of pure corundum and placed in a corundum crucible in a Netzsch STA 409 TG/DTA device. The continuous air flow rate was 75 mL/min. The heating rate was set to 10 K/min, starting from room temperature up to 1250 °C. All samples were dried to mass constancy at 120 °C prior to thermal analyses.

Complete transformation to the α-Al_2_O_3_ phase and phase purity were confirmed by X-ray diffraction on a D8 Discover by Bruker, using a Vantec500-2D detector.

## Results and Discussion

**Figure 1:**
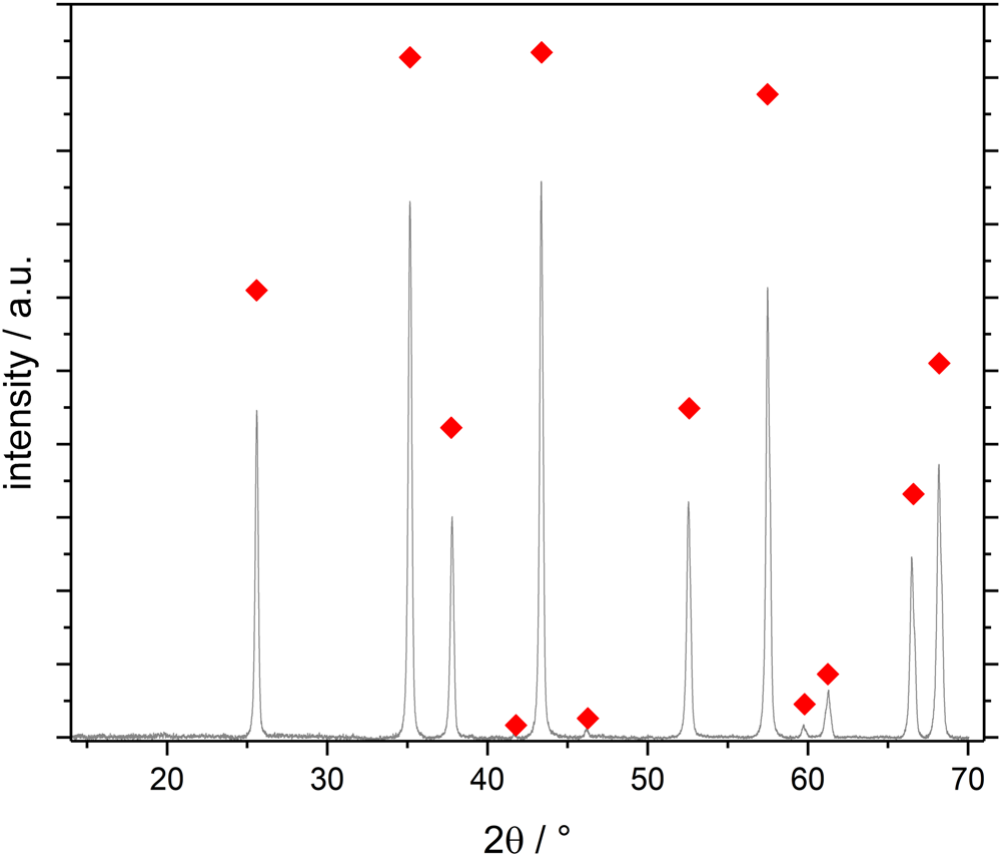
X-ray diffractogram of sample C3 after calcination at 1200 °C for 6 h confirms complete transformation to α-Al_2_O_3_ (). All other samples display the same diffraction pattern.

Depending on preparation conditions and thermal history, alumina can be generated in different modifications. The thermodynamically stable, intrinsically almost non-porous one is α-Al_2_O_3_, which is obtained from dried gels by calcination at 1200 °C for 6 h. We confirmed complete transformation to α-Al_2_O_3_ for all synthesized samples by XRD to ensure that none of the generated porosity is due to remaining fractions of transition alumina, which is often the case even in patented processes^17^. An exemplary diffractogram of sample C3 is shown in Fig. 1.

By adding dicarboxylic acids to the standard sol-gel assay, porosity can be enhanced significantly. The additive-free sample *Ref0* exhibits pores of 116 nm width, accumulating to a pore volume *V*_*p*_ of 0.12 cm^3^/g. Only marginal deviations thereof are observed when 10 mol-% of adipic acid (sample C6, *φ*_*Al*_ = 10) are added. However, these values can be augmented up to *d*_*p*_ = 838 nm, and *V*_*p*_ = 1.18 cm^3^/g, by adding the same equivalent of oxalic acid (sample C2#1, cf. Table 2). Porosity figures for malonic acid (C3), succinic acid (C4), and glutaric acid (C5) steadily decrease from these peak values down to the ones obtained for C6 and *Ref0*, as listed in Table 2. The virtual absence of porosity enhancement for C6 can partly be attributed to the poor solubility of adipic acid in water and ethanol. (Dissolution of the additive in the reaction mixture took several hours. We hence refrained from pursuing assays with dicarboxylic acids C7 and higher homologues.) More importantly, however, the general pore widening mechanism by DCA addition in the sol-gel process needs to be elucidated to explain the observed microstructural characteristics.

**Table 2:** Textural properties of all synthesized gels after calcination at 1200 °C for 6 h. Samples *Ref0*, *CA68*, *CA34*, and *CA27* are adapted from^16^.

sample	additive	*φ*_*Al*_^[a]^	*V*_*p*_^[b]^/cm^3^/g	*d*_*p*_^[b]^ / nm	*A*_*BET*_^[c]^/m^2^/g
*Ref0*^16^	*none*	*n/a*	*0.12*	*116*	*5*
*CA68*^16^	*citric acid*	10	*1.19*	*157; 3730*	6
*CA34*^16^	*citric acid*	20	*0.79*	916	6
*CA27*^16^	*citric acid*	25	*0.67*	326	9
C2#1	oxalic acid	10	1.18	838	n.d.
C2#2	oxalic acid	5	1.53	147; 2530	5
C3	malonic acid	10	0.75	259	10
C4	succinic acid	10	0.43	182	8
C5	glutaric acid	10	0.38	195	n.d.
C6	adipic acid	10	0.18	138	n.d.

^[a]^Ratio Al^3+^/additive.

^[b]^Calculated from mercury intrusion.

^[c]^Calculated from nitrogen sorption.

^n.d^not determined.

**Figure 2:**
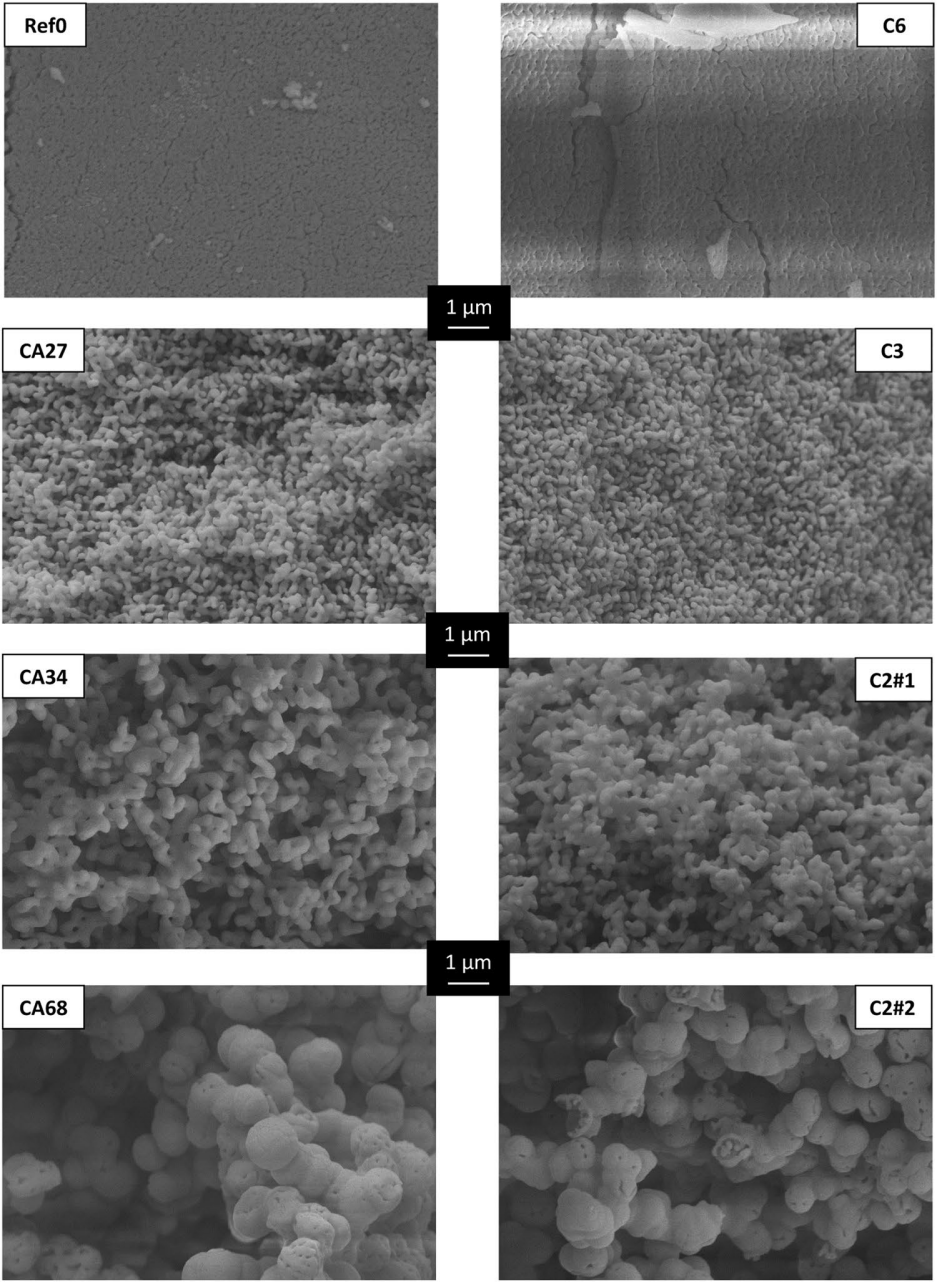
SEM images of samples *Ref0*, *CA27*, *CA34*, and *CA68* are adapted from^16^. SEM images of samples C2#1, C3, and C6 illustrate the increase of pore diameter and pore volume with decreasing DCA chain length. Sample C2#2 with doubled amount of additive exhibits phase separation. Juxtaposition of samples *Ref0* and C6, *CA27* and C3, *CA34* and C2#1, and *CA68* and C2#2, respectively, shows virtually identical porosities via different approaches, while pore volumes are consistently larger with DCA templating. All samples were calcined at 1200 °C for 6 h.

Clearly the enhancing effect on porosity is strongest for the shortest DCA, oxalic acid. In our recently published article on citric acid-assisted synthesis of highly porous α-Al_2_O_3_, we provided evidence for the formation of citrate-Al(III)-oligomers, eventually leading to phase separation and thereby an increased porosity^16^. For DCAs, the same underlying mechanism can be postulated, which is illustrated by SEM images given in Fig. 2.

**Figure 3:**
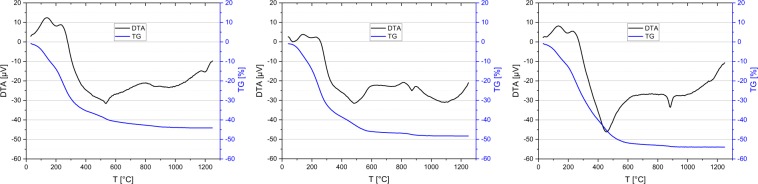
Thermal Analyses (TG/DTA) of as-synthesized dried gels *Ref0-120* (left), C3-120, containing malonic acid (center), and *CA68-120*, containing citric acid (right). Graphs for *Ref0-120* and *CA68-120* are adapted from ^16^.

Comparative thermal analyses of samples C3, *CA68*, and the additive-free reference *Ref0* support this interpretation, as they reveal complete incorporation of malonic acid into the alumina network. No discrete mass loss for the DCA is visible in the TG curve in Fig. 3. However, the total weight loss amounts to 44.1 % for sample *Ref0*, while C3 presents a weight loss of 48.3 %, hence including the 0.34 g of malonic acid. Correspondingly, the weight loss amounts to 54.0 % for sample *CA68*, containing 0.68 g of citric acid. (The difference to pure Al(OH)_3_ as starting material, which would result in a weight loss of only 35 %, can be explained by physisorbed excess solvent for all three samples.) Moreover, just like previously observed on sample *CA68*, the endothermic phase transition peak at 535 °C is missing in the DTA curve of sample C3. This indicates the strong structure directing effect of malonic acid, preventing the formation of a transitional γ-phase, and provides evidence of the incorporation of malonic acid into the alumina network on a molecular level. (For a more detailed discussion of the mechanism, please refer to ^16^).

Addition of adipic acid then leads to almost no perceptible change in microstructure and porosity, since adipate-Al(III)-oligomers with their aliphatic C4-units are poorly soluble in the strongly polar medium and thus will not grow extensively to form larger particles. A shorter DCA chain facilitates the solubility of forming dicarboxylate-Al(III)-oligomers in a polar medium like the employed reaction mixture. The oligomers hence grow to form larger particles, resulting in a larger volume fraction of nanoparticle-free solvent, which in turn not only yields larger pore diameters but also an increased pore volume. This effect can be seen by comparing the SEM images of C3 and C2#1. Finally, when increasing the DCA concentration, phase separation occurs just like for citric acid-assisted samples. To demonstrate this effect, an additional sample C2#2 was prepared with twice the amount of oxalic acid as in sample C2#1, leading to *φ*_*Al*_ = 5. The excess oxalic acid then causes the growth of larger primary alumina particles and consequently an eventual enthalpy-driven phase separation, in analogy to the one observed for citric acid^16^. Juxtaposition of samples *CA68* and C2#2 in Fig. 2 shows the almost identical microstructural habitus. Further increase of the added amount of oxalic acid is not possible due to its water solubility of only 90–100 g/L for the non-hydrated form. It would be feasible for malonic acid since its solubility in water is 15 times higher^18^. However, due to the additional carbon atom in the chain, the generation of larger pores like in sample C2#2 would require the addition of vast amounts of malonic acid.

**Figure 4:**
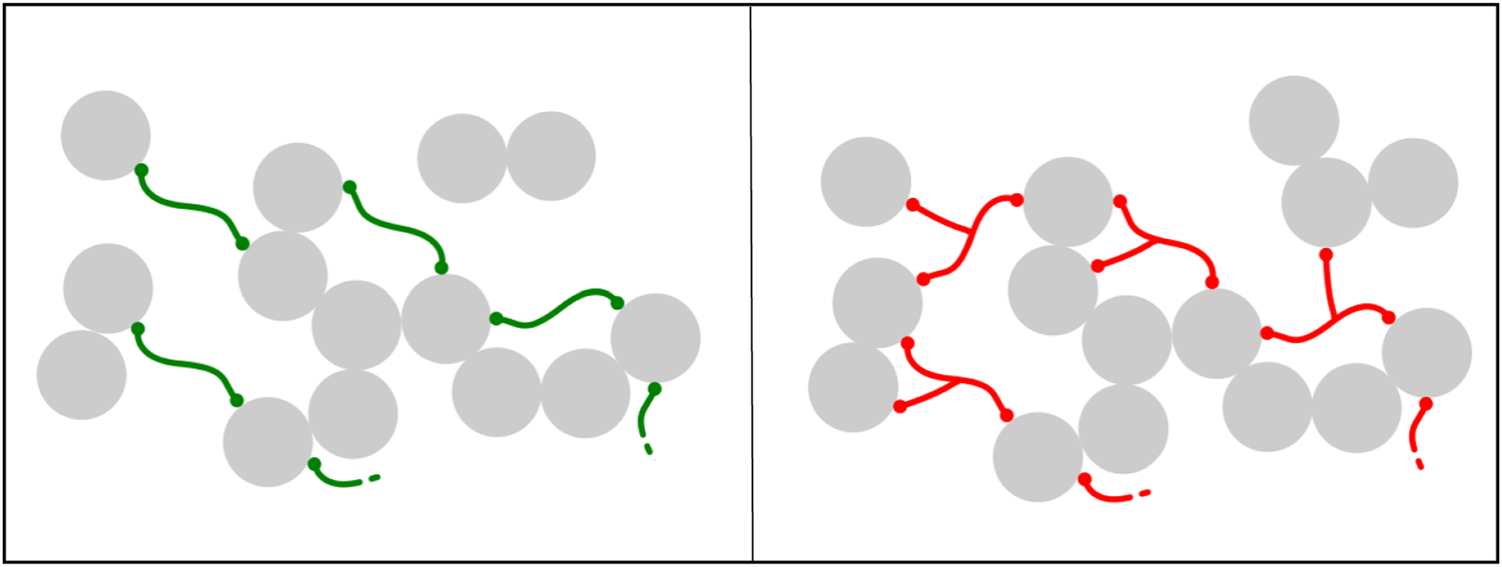
Schematic drawing of alumina sol particles (before gelation) with different additives. Individual alumina nuclei (represented by gray circles) containing one or multiple Al(III)-ions are linked via oxo- or hydroxo-bridging. Linear dicarboxylic acids (left, green) form smaller particles than the same amount of citric acid does (right, red), since the latter one is a branched molecule. (Dots represent terminal carboxylic acid groups coordinated to alumina nuclei).

**Figure 5:**
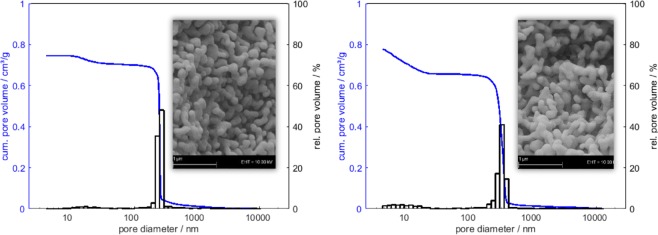
Pore size distributions of samples C3 (left) and corresponding citric acid-templated sample CA27 (right) determined by mercury intrusion. The insets show close-up SEM images depicting the monomodal macropore structure.

Despite the closely related mechanism, there is one major difference between the branched tricarboxylic citric acid and the linear dicarboxylic acids presented in this article. The latter ones offer no branching opportunities via the organic linkers, as illustrated in Fig. 4. Particles hence grow more uniformly, resulting in a more homogeneous alumina network, as shown in Figs. 2 and 5 (close-ups). The mercury intrusion histogram of sample C3 depicts a very narrow monomodal pore width distribution, with ≈ 80 % of the pores exhibiting the modal diameter. Citric acid is a branched molecule and thus can link more alumina nuclei, as schematically shown in Fig. 4. *CA27* primary particles hence grow to about the same size as for C3 with only 60 % of the carboxylic acid functional groups.

The effect of this unbranched linking in DCA is reflected in larger *V*_*p*_ generated for relatively smaller *d*_*p*_ with DCA. For instance, oxalic acid, added with ratio of *φ*_*Al*_ = 10 (sample C2#1) yields a *V*_*p*_ of 1.18 cm^3^/g, with a *d*_*p*_ of only 838 nm, while for an identical pore volume, CA addition (sample *CA68*, also with *φ*_*Al*_ = 10) yields a *d*_*p*_ of 3730 nm (cf. Table 2). As a second example, comparison of samples C3 (*φ*_*Al*_ = 10) and *CA34* (*φ*_*Al*_ = 20) reveals the impact on the resulting *A*_*BET*_. For a slightly smaller *V*_*p*_ (0.75 vs. 0.79 cm^3^/g), sample C3 exhibits an *A*_*BET*_ of 10 m^2^/g due to its smaller *d*_*p*_ of inly 259 nm, while for sample *CA34*, larger pores (*d*_*p*_ = 916 nm) lead to a reduced *A*_*BET*_ of 6 m^2^/g (cf. Table 2).

Moreover, CA already induces phase separation at *φ*_*Al*_ = 15. Thus, the generation of a controlled monomodal pore system is considerably more difficult than with dicarboxylic acids, since small deviations in the amount of additive entail a significant alteration of the resulting porosity. The use of DCA thus broadens the range of attainable pore diameters, with both monomodal pore size distributions and enhanced pore volumes, offering new opportunities in terms of their use as catalyst support material, for instance^4^.

Apart from the limited solubility of DCA with even carbon chain numbers and their tendency to esterify with solvent alcohols, there are no major disadvantages to the DCA route. On the contrary, the two major improvements using DCA as templates in comparison with citric acid consist in increased pore volumes and more uniform monomodal pore structures. Sample C3, for instance, is the one with the highest BET surface area of all DCA-templated α-Al_2_O_3_ gels due to its drastically increased pore volume in combination with a still relatively small pore diameter, which is yet sufficiently large to withstand sintering at higher temperatures ( < 1350 °C). This renders DCA-templated α-Al_2_O_3_ particularly apt for further high temperature applications in refractory ceramic foams^4^, which then may be used as catalyst supports in catalytic cracking, for instance^17,19^.

## Conclusion

Dicarboxylic acids were employed for the first time as porogenes in the sol-gel synthesis of macroporous α-Al_2_O_3_. The corresponding mechanism likely proceeds via dicarboxylate-Al(III)-oligomers, which yield larger primary particles, and consequently result in larger pore widths. With increasing carbon chain length, this effect decreases and almost vanishes for adipic acid, which forms only small linked structures, since larger ones would be insoluble.

Best results in adjusting pore diameters and increasing pore volumes are obtained for oxalic and malonic acid, leading to very narrow monomodal pore structures with pore sizes in the range of 200 to 900 nm (for 10 mol-% of DCA). When increasing the amount of additive, phase separation can be induced, resulting in similar structures as the ones obtained for citric acid, with pore diameters going up to 2.5 µm. However, larger amounts of DCA are necessary to attain the same effect as with the branched citric acid, which can link more alumina nuclei with its additional carboxylic acid group. In summary, dicarboxylic acids enable a more precise control of monomodal pore size and the generation of higher pore volumes in macroporous α-Al_2_O_3_ synthesized via the sol-gel process than the previously reported routes involving citric acid or PEO.

## Data Availability

The datasets generated during and/or analyzed during the current study are available from the corresponding author on reasonable request.
